# Prevalence and predictors of suicidality and non‐suicidal self‐harm among individuals at clinical high‐risk for psychosis: Results from a community‐recruited sample

**DOI:** 10.1111/eip.13075

**Published:** 2020-12-28

**Authors:** Kate Haining, Olga Karagiorgou, Ruchika Gajwani, Joachim Gross, Andrew I. Gumley, Stephen M. Lawrie, Matthias Schwannauer, Frauke Schultze‐Lutter, Peter J. Uhlhaas

**Affiliations:** ^1^ Institute of Neuroscience and Psychology, University of Glasgow Scotland UK; ^2^ Institute of Health and Wellbeing, University of Glasgow Scotland UK; ^3^ Department of Psychiatry University of Edinburgh Edinburgh Scotland UK; ^4^ Department of Clinical Psychology University of Edinburgh Edinburgh Scotland UK; ^5^ Department of Psychiatry and Psychotherapy, Medical Faculty Heinrich Heine University Düsseldorf Germany; ^6^ Department of Psychology and Mental Health, Faculty of Psychology Airlangga University Surabaya Indonesia; ^7^ University Hospital of Child and Adolescent Psychiatry and Psychotherapy University of Bern Bern Switzerland; ^8^ Department of Child and Adolescent Psychiatry Charité Universitätsmedizin Berlin Germany

**Keywords:** clinical high‐risk, first‐episode psychosis, psychosis, suicidality, self‐harm

## Abstract

**Aim:**

Suicidal thoughts and behaviours are prevalent in individuals with schizophrenia. However, research examining the prevalence and predictors of suicidality and self‐harm in participants at clinical high‐risk for psychosis (CHR‐P) is limited and mostly focuses on help‐seeking participants recruited through clinical pathways. The current study sought to assess the prevalence of suicidality and self‐harm and identify predictors of current suicidal ideation in community‐recruited CHR‐P participants.

**Methods:**

Data were available for 130 CHR‐P participants, 15 participants with first‐episode psychosis (FEP), 47 participants not fulfilling CHR‐P criteria (CHR‐Ns) and 53 healthy controls. Current and lifetime suicidality and self‐harm were assessed using the Mini‐International Neuropsychiatric Interview and the Comprehensive Assessment of At‐Risk Mental States (CAARMS). Multivariable logistic regression analysis was used to determine predictors of current suicidal ideation in the CHR‐P group.

**Results:**

A considerable proportion of CHR‐P participants disclosed current suicidal ideation (34.6%). Overall, FEP individuals were at greatest risk, with considerably high prevalence rates for current suicidal ideation (73.3%), lifetime self‐harm behaviour (60.0%) and lifetime suicide attempt (60.0%). In the CHR‐P sample, current suicidal ideation was predicted by lifetime suicide attempts, lower CAARMS severity, impaired social functioning and greater comorbidity.

**Conclusions:**

Our findings suggest that suicidality and self‐harm are highly prevalent in community‐recruited CHR‐P and FEP individuals. Accordingly, these results highlight the importance of further research into the determinants of suicidality and self‐harm during at‐risk and early stages of psychosis, and the implementation of intervention strategies to reduce adverse outcomes in these populations.

## 1 INTRODUCTION

Psychotic disorders, such as schizophrenia, are strongly linked to high levels of suicidality. Compared to the general population, individuals with schizophrenia have a 13‐fold greater risk of suicide (Too et al., 2019) and approximately 4.9% die by suicide (Palmer et al., 2005). Individuals with first‐episode psychosis (FEP) comprise a particularly vulnerable group. Indeed, suicide risk is elevated by 60% within the first year of treatment relative to later stages (Nordentoft et al., 2004).

Research examining the prevalence of suicidality and self‐harm in individuals at clinical high‐risk for psychosis (CHR‐P) is more limited albeit emerging (L. Pelizza et al., 2020; Taylor et al., 2015). CHR‐P participants are characterised using ultra‐high risk (UHR) criteria, which include attenuated psychotic symptoms, brief frank psychosis and functional decline with genetic risk (Yung et al., 2005), as well as basic symptom criteria relying on perceptual and cognitive disturbances self‐experienced with full and immediate insight (F. Schultze‐Lutter, 2009; F. Schultze‐Lutter et al., 2012). Over a 2‐year period, around 20% of individuals meeting UHR criteria transition to psychosis (P. Fusar‐Poli, Cappucciati, et al., 2016). Moreover, in a UHR sample, approximately 45% of nonconverters experienced either poor social or role outcome (Carrión et al., 2013); impairments previously related to persistence of CHR‐P symptoms (Michel et al., 2018).

A recent meta‐analysis reported prevalence rates of 66% for current suicidal ideation, 18% for lifetime suicide attempts and 49% for lifetime self‐harm behaviour in UHR samples, comparable to those observed in FEP cohorts (Taylor et al., 2015).Furthermore, in a retrospective study of prodromal suicide risk among individuals with schizophrenia, 25.5% had experienced suicidal ideation and 7.5% had attempted suicide (Andriopoulos et al., 2011). More recently, L. Pelizza et al. (2020) found that UHR individuals disclosed more severe suicidal ideation and were more likely to report previous suicide attempts than FEP and non‐UHR/FEP samples. Therefore, there is a need to further identify the factors underlying the emergence of suicidality and self‐harm in CHR‐P populations.

However, relatively little is known about the predictors of suicidality and self‐harm in CHP‐P individuals. Paranoid thinking, depressive symptoms and impaired role functioning have been found to predict current suicidal ideation (Andriopoulos et al., 2011; Bang et al., 2017; L. Pelizza et al., 2019), while the presence of personality disorders and history of trauma strongly predict suicide attempts (Zuschlag et al., 2018), consistent with findings in established schizophrenia and other psychiatric populations (Aaltonen et al., 2016; Bornheimer, 2016; Fuller‐Thomson & Hollister, 2016).Within these latter cohorts, suicidal ideation and previous suicide attempts have been identified as two of the strongest predictors of completed suicide (Fosse et al., 2017; Lopez‐Morinigo et al., 2016) and future suicide attempts (Bertelsen et al., 2007; Horwitz et al., 2015).

To date, the majority of studies investigating suicidality and self‐harm in CHR‐P populations involve help‐seeking participants recruited through clinical pathways by UHR criteria. Accordingly, it is unclear whether the prevalence rates and predictors of suicidality and self‐harm identified in these studies generalise to more representative community samples as well as CHR‐P individuals recruited using UHR and/or basic symptom criteria. This is an important question given that recruitment pathways have been shown to impact on transition rates in CHR‐P samples. Indeed, pretest risk for psychosis, although enriched in help‐seeking samples, appears to be lower in community‐recruited samples, reducing the likelihood of subsequent transitions (P. Fusar‐Poli, Schultze‐Lutter, et al., 2016).

In the current study, we sought to assess the prevalence of suidicality and self‐harm in community‐recruited CHR‐P and FEP participants. We also included participants who did not fulfil CHR‐P criteria but were characterised by psychiatric comorbidities (CHR‐Ns) as well as a group of healthy controls (HCs). In addition, we aimed to identify predictors of current suicidal ideation in the CHR‐P group. Social support, insecure attachment orientations and cognitive ability were also investigated given their relation with suicidality in the general population (E.M. Kleiman & Liu, 2013; Kosidou et al., 2014; Sörberg et al., 2013; Zortea et al., 2019).

Given these findings, we hypothesised that (1) CHR‐P and FEP participants would show comparably higher levels of suicidality and self‐harm than CHR‐N and HC participants and (2) a range of clinical, functional and cognitive variables would emerge as significant predictors of current suicidal ideation in CHR‐P participants.

## 2 METHODS

### 2.1 Participants

Participants were recruited as part of the Youth Mental Health Risk and Resilience (YouR) study(Uhlhaas et al., 2017), an ongoing longitudinal study funded by the Medical Research Council which aims to identify neurobiological and psychological mechanisms and predictors of psychosis risk. Utilising an online‐screening approach (McDonald et al., 2019), potential CHR‐P participants from the general population were directed to our website (www.yourstudy.org.uk) via email invitations, posters and flyers over a 4‐year period. FEP and CHR‐N participants were also recruited using this approach while HCs were obtained from an existing volunteer database. Screening questionnaires comprised (a) the 16‐item Prodromal Questionnaire (PQ‐16; Ising et al., 2012) and (b) a nine‐item scale of Perceptual and Cognitive Anomalies (PCA) for assessing basic symptoms. Participants were invited for clinical interviews if they positively endorsed six or more items on the PQ‐16 and/or three or more items on the PCA.

Data were available for 130 CHR‐P individuals that were recruited across two sites: Glasgow (*n* = 94; 72.3%) and Edinburgh (*n* = 36; 27.7%).We also obtained a community‐recruited sample of 15 FEP participants, 47 CHR‐N participants and 53 HCs.

### 2.2 Instruments and measures

In order to establish CHR‐P criteria, the positive scale of the Comprehensive Assessment of At‐Risk Mental States (CAARMS; Yung et al., 2005) and the Cognitive Disturbances (COGDIS) and Cognitive‐Perceptive Basic Symptoms (COPER) items of the Schizophrenia Proneness Instrument, Adult version (SPI‐A; F. Schultze‐Lutter et al., 2007) were administered by trained research assistants and MSc/PhD level researchers. Participants were recruited into the CHR‐P group if they met SPI‐A COGDIS/COPERcriteria and/or one of the following CAARMS criteria: attenuated psychotic symptoms (APS), genetic risk and functional deterioration (GRFD) or brief limited intermittent psychotic symptoms. FEP criteria were established using the Positive and Negative Syndrome Scale (Kay et al., 1987) as well as the Structured Clinical Interview for DSM‐IV (SCID; First et al., 2002).

Current (past month) and lifetime suicidality and self‐harm were assessed using the six‐item suicidality module of the Mini‐International Neuropsychiatric Interview (MINI; Sheehan et al., 1998) as well as questions contained in the CAARMS suicidality and self‐harm subscale. For FEP participants, only the latter assessment of suicidality and self‐harm was available.

In addition, with the exception of the FEP group, all participants were assessed with the Global Functioning: Social (GF: Social) and Role (GF: Role) scales (Cornblatt et al., 2007), Premorbid Adjustment Scale (Cannon‐Spoor et al., 1982), Adverse Childhood Experiences Scale (Felitti et al., 1998), Psychosis Attachment Measure (Berry et al., 2006), Significant Others Scale (Power et al., 1988)and Brief Assessment of Cognition in Schizophrenia (BACS; Keefe et al., 2004). Psychiatric comorbidity was calculated from the MINI by summing the number of current comorbid Axis I disorders disclosed by participants from a possible total of five (mood disorder, anxiety disorder, drug abuse/dependence, alcohol abuse/dependence, eating disorder).

### 2.3 Statistical methods

Data were analysed using SPSS version 26 with statistical significance set at *p* < .05 (two‐tailed). The BACS composite score was calculated by averaging the z‐scores obtained from the six primary measures and re‐standardizing this value using the means and standard deviations of sex‐specific HCs (Keefe et al., 2004). Overall, 1.2% of the data (48 of 4,030 values) were missing and imputed by Bayesian imputation.

Group differences were analysed using non‐parametric Kruskal–Wallis *H* tests and chi‐square tests followed by appropriate Bonferroni‐corrected post hoc tests if required. Collinearity of predictors was defined as any variance inflation factor (VIF) > 2 and tolerance <0.40. Multivariable logistic regression analysis, using stepwise backward selection (likelihood ratio), was employed to determine predictors of current suicidal ideation in the CHR‐P group. This outcome variable was prioritised as it did not violate the events per variable rule of 5:1 suggested by Vittinghoff and McCulloch (2007). The overall variance explained by the model was measured by the Nagelkerke pseudo R^2^ statistic (R^2^N). Diagnostic accuracy of the model was determined using the area under the receiver‐operating characteristic curve (AUC).

## 3 RESULTS

### 3.1 Demographic data

CHR‐P individuals were significantly impaired relative to CHR‐N and HC participants, displaying greater CAARMS and SPI‐A severity, higher comorbidity, lower social and role functioning and greater levels of insecure attachment (Table 1). As expected, FEP participants had significantly higher CAARMS severity compared to all other groups and greater antipsychotic use compared to CHR‐P participants. Although significant group differences emerged for age, these effects did not survive Bonferroni‐corrected post hoc tests.

**TABLE 1:** Demographic, clinical, functional and cognitive characteristics of the total sample (N = 245)

	CHR‐P (1) (N = 130)	FEP (2) (N = 15)	CHR‐N (3) (N = 47)	HC (4) (N = 53)	P	Effect size^a^	Post hoc test^b^
Age (years), mean (SD)	21.64 (4.27)	23.73 (4.79)	22.94 (4.80)	22.42 (3.36)	.044	η^2^_*p*_ = 0.033	··
Gender, female n (%)	94 (72.3)	10 (66.7)	30 (63.8)	36 (67.9)	.727	V = 0.073	··
Education (years), mean (SD)	15.40 (2.95)	15.80 (3.38)	16.45 (3.44)	16.47 (2.85)	.070	η^2^_*p*_ = 0.029	
Suicidality and self‐harm, n (%)							
*Self‐harm intention (past month)*	37 (28.5)	··	4 (8.5)	0 (0)	<.001	V = 0.325	1 > 3,4
*Self‐harm behaviour (past month)*	7 (5.4)	3 (20.0)	0 (0)	0 (0)	.005	V = 0.244	2 > 3,4
*Self‐harm behaviour (lifetime)*	37 (28.5)	9 (60.0)	5 (10.6)	2 (3.8)	<.001	V = 0.349	2 > 3,4 & 1 > 4
*Suicide plan (past month)*	12 (9.2)	1 (6.7)	3 (6.4)	1 (1.9)	.332	V = 0.114	··
*Suicidal ideation (past month)*	45 (34.6)	11 (73.3)	9 (19.1)	1 (1.9)	<.001	V = 0.397	2 > 1,3,4 & 1,3 > 4
*Suicide attempt (past month)*	3 (2.3)	1 (6.7)	0 (0)	0 (0)	.201	V = 0.134	··
*Suicide attempt (lifetime)*	38 (29.2)	9 (60.0)	4 (8.5)	0 (0)	<.001	V = 0.393	1,2 > 3,4
MINI suicidality risk, n (%)							
*Low*	28 (21.5)	··	3 (6.4)	1 (1.9)	.001	V = 0.255	1 > 4
*Moderate*	21 (16.2)	··	3 (6.4)	0 (0)	.003	V = 0.224	1 > 4
*High*	21 (16.2)	··	5 (10.6)	0 (0)	.007	V = 0.207	1,3 > 4
CAARMS severity, median (range)	29 (0‐74)	88 (38‐122)	6 (0‐24)	0 (0‐12)	<.001	η^2^_*p*_ = 0.408	2 > 1 > 3 > 4
SPI‐A severity, median (range)	7 (0‐74)	14 (0‐109)	0 (0‐7)	0 (0‐2)	<.001	η^2^_*p*_ = 0.338	1, 2 > 3,4
ACES total, median (range)	2 (0‐8)	··	1 (0‐5)	0 (0‐4)	<.001	η^2^_*p*_ = 0.111	1 > 4
Comorbidity, median (range)	2 (0‐5)	··	1 (0‐3)	0 (0)	<.001	η^2^_*p*_ = 0.306	1,3 > 4 & 1 > 3
Psychological treatment, n (%)							
*Current*	21 (16.2)	3 (20.0)	5 (10.6)	0 (0)	.015	V = 0.207	1,2 > 4
*Past*	59 (45.4)	9 (60.0)	15 (31.9)	3 (5.7)	<.001	V = 0.353	1,2,3 > 4
Medication, n (%)							
*Antidepressants*	46 (35.4)	7 (46.7)	13 (27.7)	0 (0)	<.001	V = 0.333	1,2,3 > 4
*Mood stabilisers*	4 (3.1)	0 (0)	0 (0)	0 (0)	.534	V = 0.121	··
*Antipsychotics*	2 (1.5)	2 (13.3)	0 (0)	0 (0)	.039	V = 0.243	2 > 1,4
*Anxiolytics*	8 (6.2)	2 (13.3)	1 (2.1)	0 (0)	.060	V = 0.165	··
Social functioning (current), median (range)	8 (3‐10)	··	8 (6‐9)	9 (8‐10)	<.001	η^2^_*p*_ = 0.224	4 > 1,3 & 3 > 1
Role functioning (current), median (range)	8 (3‐9)	··	8 (5‐9)	9 (5‐9)	<.001	η^2^_*p*_ = 0.191	4 > 1,3 & 3 > 1
PAS average, median (range)	1.20 (0‐3.43)	··	0.86 (0‐3.86)	0.43 (0‐1.64)	<.001	η^2^_*p*_ = 0.183	1,3 > 4
Social support, mean (SD)	5.05 (0.89)	··	5.30 (0.87)	6.02 (0.59)	<.001	η^2^_*p*_ = 0.168	4 > 1,3
Insecure attachment, mean (SD)	1.75 (0.46)	··	1.41 (0.50)	1.01 (0.46)	<.001	η^2^_*p*_ = 0.226	1,3 > 4 & 1 > 3
BACS composite score, mean (SD)	‐0.39 (1.64)	··	‐0.02 (1.38)	0 (1.01)	.140	η^2^_*p*_ = 0.017	··

*Note*: CHR‐P, clinical high‐risk for psychosis; FEP, first episode psychosis; CHR‐N, clinical high‐risk‐negative; HC, healthy control; MINI, Mini‐International Neuropsychiatric Interview; CAARMS, Comprehensive Assessment of At‐Risk Mental States; SPI‐A, Schizophrenia Proneness Instrument, Adult version; ACES, Adverse Childhood Experiences Scale; PAS, Premorbid Adjustment Scale; BACS, Brief Assessment of Cognition in Schizophrenia.

^a^ Effect sizes were eta squared (η^2^_*p*_) for Kruskal‐Wallis H tests (small effect = 0.01, medium effect = 0.06, large effect = 0.14) and Cramer's V for Pearson’s chi‐square or Fisher‐Freeman‐Halton tests (small effect = 0.1, medium effect = 0.3, large effect = 0.5).

^b^ 1 = CHR‐P, 2= FEP, 3 = HC, 4 = CHR‐N

A significantly larger proportion of FEP and CHR‐P participants received current or past treatment compared to HCs (Table 1). Twenty percent of FEP participants and 16.2% of CHR‐P participants were in current treatment while 60.0% of FEP participants and 45.4%of CHR‐P participants received past treatment. CHR‐N participants (31.9%) were also significantly more likely than HCs (5.7%) to have engaged in past treatment.

In addition, among the CHR‐P group, 39 (30.0%) met CAARMS criteria, 32 (24.6%) met SPI‐A criteria and 59 (45.4%) met both. Of those meeting CAARMS, 95.9% met APS criteria, 2.0% met GRFD criteria and 2.0% met both APS and GRFD criteria; while, of those meeting SPI‐A criteria, 46.2% met COPER criteria, 14.3% met COGDIS criteria and 39.6% met both. Furthermore, the FEP group consisted of participants with SCID DSM‐IV psychotic disorder not otherwise specified (n = 7; 46.7%), schizophrenia (n = 6; 40.0%) and schizoaffective disorder (n = 2; 13.3%).

### 3.2 Suicidality and self‐harm prevalence

**FIGURE 1:**
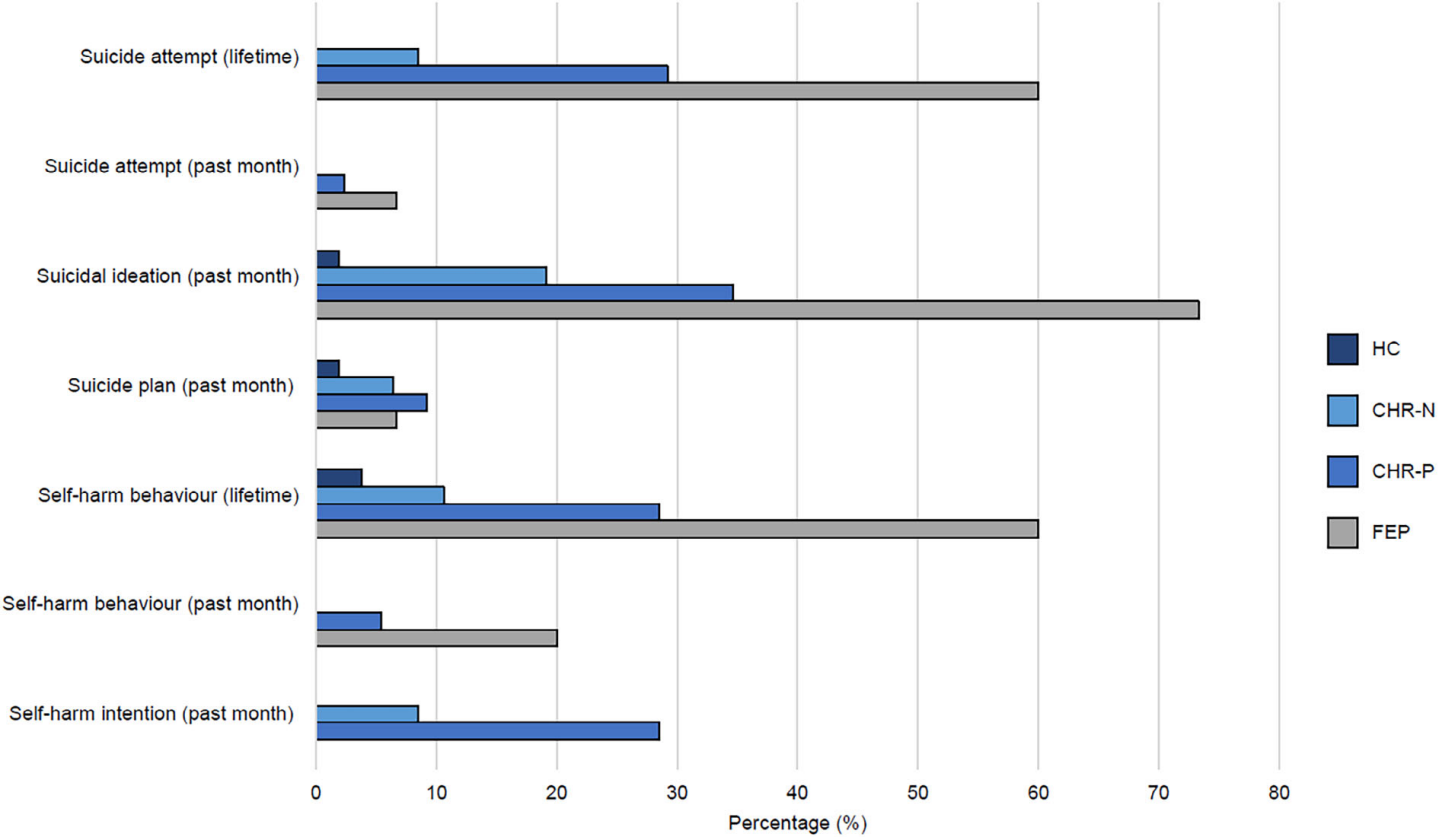
Suicidality and self‐harm profile of the total sample (*N* = 245)

Lifetime suicide attempts were significantly more prominent in individuals meeting CHR‐P (29.2%) and FEP (60.0%) criteria compared to CHR‐N (8.5%) and HC (0%) participants (Table 1; Figure 1). In addition, relative to HCs, CHR‐P participants more commonly disclosed current suicidal ideation (34.6%), current self‐harm intention (28.5%) and lifetime self‐harm behaviour (28.5%) whilst CHR‐N participants were more likely to report current suicidal ideation (19.1%). Current self‐harm intention was also reported significantly more in CHR‐P than in CHR‐N individuals (28.5 vs. 8.5%). Overall, 32.4% of CHR‐P and 17.0% of CHR‐N participants were categorised as currently at moderate‐ to high‐risk of suicide. The FEP group was at greatest risk, with considerably high prevalence rates for current suicidal ideation (73.3%), lifetime self‐harm behaviour (60.0%) and lifetime suicide attempt (60.0%).

### 3.3 Impact of recruitment pathway

We further compared our community‐recruited CHR‐P sample to a smaller group of CHR‐P individuals (*n* = 16) recruited via referrals from clinical services in NHS Greater Glasgow and Clyde and NHS Lothian as well as student counselling services (Table S1). Referred participants had significantly fewer years of education, poorer functioning and lower BACS composite score than community‐recruited participants. However, no significant group differences were observed on suicide‐related variables.

### 3.4 Predictors of current suicidal ideation in CHR‐P participants

Multivariable logistic regression analysis was used to determine predictors of current suicidal ideation in CHR‐P individuals. We did not identify any sources of multicollinearity among the potential predictor variables.

**FIGURE 2:**
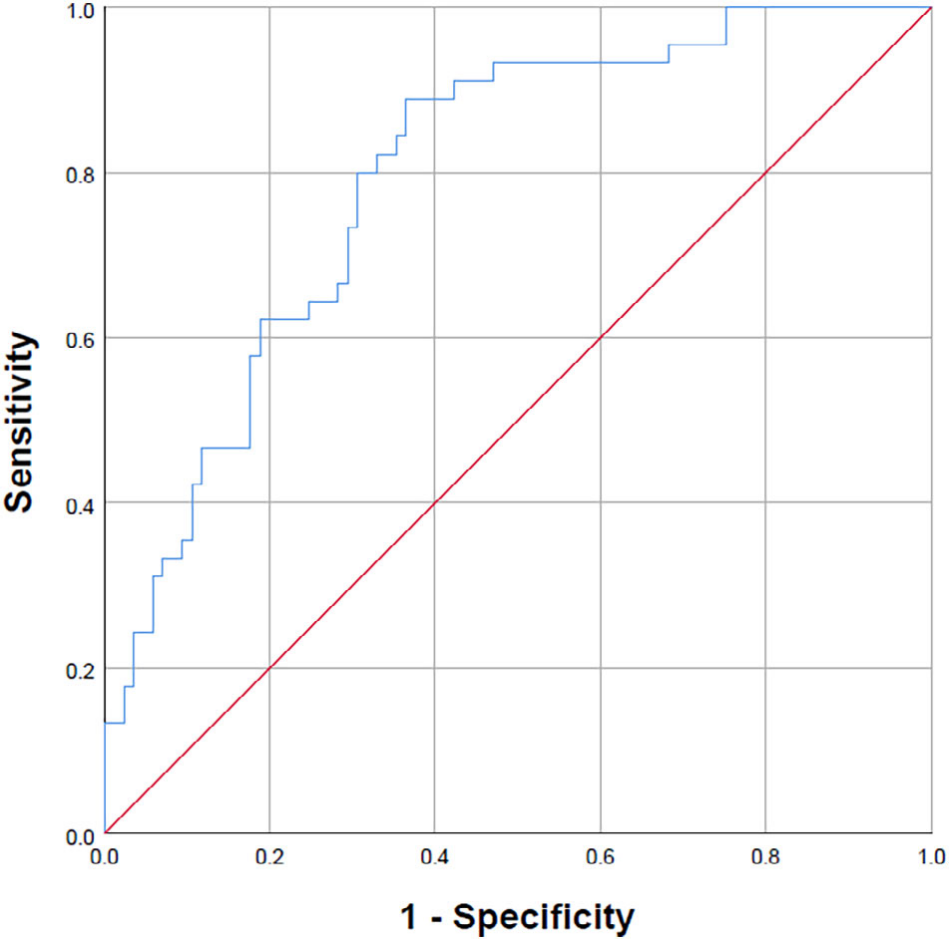
Receiver‐operating characteristic curve for the multivariable logistic regression model predicting suicidal ideation (past month) in clinical high‐risk for psychosis participants (*N* = 130)

Predictors of current suicidal ideation in CHR‐P participants included lifetime suicide attempts, lower CAARMS severity, impaired social functioning and premorbid adjustment and greater comorbidity although premorbid adjustment did not contribute significantly to the model (Table 2). This model explained 32.4% of the variance with an acceptable AUC of 0.797 (*p* < .001), specificity of 82.4% and sensitivity of 46.7% (Figure 2).

**TABLE 2:** Multivariable logistic regression model for suicidal ideation (past month) in CHR‐P participants (*N* = 130)

Variable	Beta	SE	Wald	*p*	OR (95% CI)	AUC (SE) [95% CI]	*R* ^2^_N_	Sensitivity	Specificity
Suicide attempt (lifetime)	0.994	0.484	4.221	.040	2.701 (1.047–6.969)				
CAARMS severity	−0.030	0.015	4.110	.043	0.971 (0.943–0.999)	0.797 (0.039) [0.720–0.874]	0.324	46.7	82.4
Social functioning (current)	−0.496	0.216	5.246	.022	0.609 (0.399–0.931)				
Premorbid adjustment	0.577	0.344	2.804	.094	1.780 (0.906–3.495)				
Comorbidity	0.489	0.199	6.030	.014	1.631 (1.104–2.411)			

*Note*: Beta, unstandardised regression coefficient.

Abbreviations: AUC, area under the curve; CI, confidence interval; OR, odds ratio; *R* ^2^_N_, Nagelkerke pseudo *R* ^2^ statistic.

## 4 DISCUSSION

We examined the prevalence of suicidality and self‐harm in CHR‐P and FEP samples as well as predictors of current suicidal ideation in CHR‐P individuals. Our findings suggest that suicidality and self‐harm are highly prevalent in community‐recruited CHR‐P and FEP groups with the latter at greatest risk. In addition, lifetime suicide attempts, lower CAARMS severity, impaired social functioning and greater comorbidity significantly predicted current suicidal ideation in CHR‐P participants.

### 4.1 Suicidality and self‐harm prevalence

Our findings highlight significant levels of suicidality and self‐harm in CHR‐P individuals recruited from the community. Current suicidal ideation was most commonly disclosed with a prevalence rate of 34.6%, comparable to previous estimates of 30% (DeVylder et al., 2012) and 42.9% (Gill et al., 2015) within help‐seeking UHR samples. Similarly large proportions of our CHR‐P sample reported lifetime suicide attempts (29.2%), lifetime self‐harm behaviour (28.5%) and current self‐harm intention (28.5%). Interestingly, prevalence estimates for lifetime suicide attempts are generally lower in help‐seeking UHR samples, ranging between 8.6 and 18% (Pelizza et al., 2019, 2020; Preti et al., 2009; Taylor et al., 2015). One possibility is that clinically‐recruited UHR participants, through their established contact with mental health services, have better coping skills in comparison to community‐recruited individuals. Overall, the current findings demonstrate that high rates of suicidality and self‐harm are not restricted to clinically recruited UHR samples.

In contrast to previous studies, our results suggest that suicidality and self‐harm are more prevalent in FEP as compared to CHR‐P participants, especially with regard to current suicidal ideation (L. Pelizza et al., 2019, 2020; Preti et al., 2009). Our FEP group exhibited prevalence rates for current suicidal ideation (73.3%), lifetime self‐harm behaviour (60.0%) and lifetime suicide attempts (60.0%) that were approximately two to 11 times greater than those typically reported in FEP samples (Bertelsen et al., 2007; Challis et al., 2013; Pelizza et al., 2020; Preti et al., 2009), possibly resulting from our focus on community‐recruitment. Indeed, given that only 20.0% of FEP participants were in current psychological treatment and 13.3% received antipsychotics, these individuals may not be receiving appropriate clinical attention and support for their heightened psychotic symptoms and associated distress, thereby increasing suicidality risk.

Notably, CHR‐N individuals were characterised by relatively modest suicidality and self‐harm, potentially attributable to the lower comorbidity and better functioning observed in this group relative to the CHR‐P sample. Significantly more CHR‐N participants reported current suicidal ideation (19.1%) compared to HCs (1.9%), however; contrasting with the higher prevalence rates of 33.3% (L. Pelizza et al., 2020) and 45% (L. Pelizza et al., 2019) reported in help‐seeking samples.

### 4.2 Predictors of current suicidal ideation in CHR‐P participants

In the CHR‐P group, significant predictors of current suicidal ideation included lifetime suicide attempts, lower CAARMS severity, impaired social functioning and greater comorbidity. The final model explained 32.4% of the variance in current suicidal ideation, in line with previous findings in help‐seeking UHR cohorts (Bang et al., 2017; L. Pelizza et al., 2019).

Our results also concur with prior research in UHR and schizophrenia samples wherein depressive mood, increased psychiatric comorbidity and poor functioning have emerged as predictors of suicidal ideation (Andriopoulos et al., 2011; Bornheimer, 2016; Harvey et al., 2018; L. Pelizza et al., 2019). Furthermore, these findings are in accordance with the interpersonal theory of suicide (Joiner, 2005; Van Orden et al., 2010) which implicates perceived alienation from, and lack of meaningful connections with, friends, family and others (i.e., thwarted belongingness). The emergence of lower, rather than higher, CAARMS severity as a significant predictor of current suicidal ideation, however, contrasts with previous findings in help‐seeking UHR samples (Bang et al., 2017).

Overall, the strongest predictor of current suicidal ideation was lifetime suicide attempts, concurring with previous findings in schizophrenia (Kim et al., 2010). Given that suicidal ideation is also highly predictive of future suicide attempts and completed suicide in both schizophrenia samples and psychiatric patient populations (Bertelsen et al., 2007; Fosse et al., 2017; Horwitz et al., 2015; Lopez‐Morinigo et al., 2016), effectively identifying CHR‐P individuals with current suicidal ideation is a critical step towards managing risk and reducing suicide deaths.

Contrary to findings from the general population (E.M. Kleiman & Liu, 2013; Kosidou et al., 2014; Sörberg et al., 2013; Zortea et al., 2019), social support, insecure attachment orientations and cognitive ability did not emerge as predictors of suicidality, perhaps owing to differing assessment measures. In addition, although characterised by excellent specificity, the prediction model yielded limited sensitivity. This issue is commonly noted for suicide prediction models, which may limit their clinical value (Kessler et al., 2020). In order to optimise model performance, future research should consider employing advanced machine learning methods as well as more comprehensive predictor sets incorporating, for example, biological predictors.

### 4.3 Limitations

The sample size of CHR‐P participants with current suicidal ideation was relatively small, limiting the number of variables that could be included in a single model and perhaps reducing the generalisability of the findings.

In addition, information regarding suicidality and self‐harm was elicited via self‐report questions—a method particularly susceptible to social desirability response bias; or to exaggeration by individuals seeking help. Our methodological approach also involved a single retrospective assessment of suicidality and self‐harm (e.g., past month/lifetime). Given that suicidal ideation is known to fluctuate rapidly over just a few hours, this approach may be of limited value (E.M. Kleiman et al., 2017). In order to capture fine‐grained variation in suicidality and self‐harm, future research should turn to time‐intensive techniques such as ecological momentary assessment which allow data to be collected repeatedly, in real‐time and in naturalistic settings (de Beurs et al., 2015).

## 5 CONCLUSIONS

Our findings emphasise the high prevalence of suicidality and self‐harm in community‐recruited CHR‐P and FEP individuals. Moreover, we demonstrated that lifetime suicide attempts, lower CAARMS severity, impaired social functioning and greater comorbidity were able to significantly predict current suicidal ideation in CHR‐P participants, with lifetime suicide attempts comprising the strongest predictor. Therefore, the current findings highlight that CHR‐P individuals recruited outside traditional early intervention services represent a vulnerable group that requires novel approaches for detection; and early intervention aimed at suicide prevention. Whether prediction models can be applied to suicidality prevention in CHR‐P samples remains, however, an open question. We expect that, by incorporating larger collaborative datasets, longitudinal study designs, machine learning approaches and real‐time measures, model performance will improve, thereby optimising youth mental health.

## CONFLICT OF INTEREST

Prof. Uhlhaas has received research support from Lilly and Lundbeck outside the submitted work.

## Supporting information

**Table S1** Demographic, clinical, functional and cognitive characteristics of CHR‐P participants by recruitment pathway (*N* = 146)

## Data Availability

The datasets generated during and/or analysed during the current study are available from the corresponding author on reasonable request.
