# Supplementary material for: Prevalence and predictors of suicidality and non‐suicidal self‐harm among individuals at clinical high‐risk for psychosis: Results from a community‐recruited sample
Source: Early Interv Psychiatry. 2020 Dec 28;15(5):1256–65. doi: 10.1111/eip.13075 (PMC8451831; doi:10.1111/eip.13075)
Supplement: Supplementary file 1 — Table S1 Demographic, clinical, functional and cognitive characteristics of CHR‐P participants by recruitment pathway (N = 146) [file EIP-15-1256-s001.docx]

**Table S1.** Demographic, clinical, functional and cognitive characteristics of CHR-P participants by recruitment pathway (N = 146)

|  | Community  (n = 130) | Referral  (n = 16) | *P* | Effect size^a^ |
| --- | --- | --- | --- | --- |
| Age (years), mean (SD) | 21.64 (4.27) | 20.06 (3.62) | .121 | r = 0.129 |
| Gender, female n (%) | 94 (72.3) | 10 (62.5) | .397 | ϕ = 0.068 |
| Education (years), mean (SD) | 15.40 (2.95) | 12.88 (3.40) | .001 | r = 0.267 |
| Suicidality and self-harm, n (%) |  |  |  |  |
| *Self-harm intention (past month)* | 37 (28.5) | 1 (6.3) | .070 | ϕ = 0.158 |
| *Self-harm behaviour (past month)* | 7 (5.4) | 0 (0) | 1.000 | ϕ = 0.079 |
| *Self-harm behaviour (lifetime)* | 37 (28.5) | 2 (12.5) | .237 | ϕ = 0.113 |
| *Suicide plan (past month)* | 12 (9.2) | 2 (12.5) | .653 | ϕ = 0.035 |
| *Suicidal ideation (past month)* | 45 (34.6) | 4 (25.0) | .442 | ϕ = 0.064 |
| *Suicide attempt (past month)* | 3 (2.3) | 0 (0) | 1.000 | ϕ = 0.051 |
| *Suicide attempt (lifetime)* | 38 (29.2) | 8 (50.0) | .092 | ϕ = 0.140 |
| MINI suicidality risk, n (%) |  |  |  |  |
| *Low* | 28 (21.5) | 5 (31.3) | .359 | ϕ = 0.073 |
| *Moderate* | 21 (16.2) | 0 (0) | .129 | ϕ = 0.144 |
| *High* | 21 (16.2) | 2 (12.5) | 1.000 | ϕ = 0.031 |
| CAARMS severity, median (range) | 29 (0-74) | 22 (11-54) | .148 | r = 0.120 |
| SPI-A severity, median (range) | 7 (0-74) | 5 (0-33) | .773 | r = 0.024 |
| Comorbidity, median (range) | 2 (0-5) | 1.5 (0-4) | .480 | r = 0.059 |
| ACES total, median (range) | 2 (0-8) | 1.5 (0-7) | .532 | r = 0.052 |
| Psychological treatment, n (%) |  |  |  |  |
| *Current* | 21 (16.2) | 4 (25.0) | .479 | ϕ =0.073 |
| *Past* | 59 (45.4) | 7 (43.8) | .901 | ϕ =0.010 |
| Medication, n (%) |  |  |  |  |
| *Antidepressants* | 46 (35.4) | 7 (43.8) | .511 | ϕ =0.054 |
| *Mood stabilisers* | 4 (3.1) | 0 (0) | 1.000 | ϕ =0.059 |
| *Antipsychotics* | 2 (1.5) | 2 (12.5) | .060 | ϕ =0.210 |
| *Anxiolytics* | 8 (6.2) | 2 (12.5) | .301 | ϕ =0.078 |
| Social functioning (current), median (range) | 8 (3-10) | 7 (5-9) | .070 | r = 0.150 |
| Role functioning (current), median (range) | 8 (3-9) | 6.5 (6-9) | .008 | r = 0.218 |
| PAS average, median (range) | 1.20 (0-3.43) | 1.61 (0.50-3.00) | .030 | r = 0.180 |
| Social support, mean (SD) | 5.05 (0.89) | 5.19 (1.21) | .501 | r = 0.056 |
| Insecure attachment, mean (SD) | 1.75 (0.46) | 1.71 (0.38) | .611 | r = 0.042 |
| BACS composite score, mean (SD) | -0.39 (1.64) | -1.79 (1.98) | .012 | r = 0.207 |

*Note*. CHR-P, clinical high-risk for psychosis; MINI, Mini-International Neuropsychiatric Interview; CAARMS, Comprehensive Assessment of At-Risk Mental States; SPI-A, Schizophrenia Proneness Instrument, Adult version; ACES, Adverse Childhood Experiences Scale; PAS, Premorbid Adjustment Scale; BACS, Brief Assessment of Cognition in Schizophrenia.

^a^ Effect sizes were Rosenthal's r for Mann-Whitney U tests and Phi (ϕ) for Pearson’s chi-square or Fisher′s exact tests (small effect = 0.1, medium effect = 0.3, large effect = 0.5).
